# Sexually dimorphic sail feathers in the Mandarin duck as a model for lifelong developmental modulation

**DOI:** 10.1038/s41598-025-20446-3

**Published:** 2025-10-21

**Authors:** Pin-Chi Tang, Hsu-Chen Cheng, Gee-Way Lin, Yung-Chih Lai, Ya-Chen Liang, Ping Wu, Tzu-Chiao Lin, Chen Siang Ng, Isheng Jason Tsai, Ang Li, Wen Hsiung Li, Chih-Feng Chen, Cheng-Ming Chuong, Randall Widelitz

**Affiliations:** 1 Department of Animal Science, National Chung Hsing University, Taichung, 402202 Taiwan; 2 The iEGG and Animal Biotechnology Center, National Chung Hsing University, Taichung, 402202 Taiwan; 3 Department of Life Sciences, National Chung Hsing University, Taichung, 402202 Taiwan; 4 Department of Pathology, Keck School of Medicine, University of Southern California, Los Angeles, CA 90033 USA; 5 School of Medicine, College of Medicine, Taipei Medical University, Taipei, 110301 Taiwan; 6 Institute of Translational Medicine and New Drug Development, China Medical University, Taichung, 404328 Taiwan; 7 Integrative Stem Cell Center, China Medical University Hospital, China Medical University, Taichung, 404327 Taiwan; 8 Institute of Molecular and Cellular Biology, National Tsing Hua University, Hsinchu, 30013 Taiwan; 9 Biodiversity Research Center, Academia Sinica, Taipei, 115201 Taiwan; 10 Department of Kinesiology, University of Texas, Arlington, TX 76019 USA

**Keywords:** Seasonal variation, Morphogenesis, Stem cells, Feather colors, Feather forms, Sex hormones, Feather cycling, Androgen, Estrogen, Morphogenesis, Pattern formation

## Abstract

Developmental processes extend beyond embryogenesis to support lifelong tissue adaptations. Avian feather follicles, with their resident stem cells and capacity for cyclic regeneration, provide a dynamic model for postnatal tissue remodeling. Here, we propose the Mandarin duck (*Aix galericulata*) as an ideal model to study lifelong developmental modulation, focusing on the sexually dimorphic “sail feather”—a secondary flight feather in males that undergoes seasonal transformation into a strikingly asymmetric, ornamented phenotype during the breeding season. We identified asymmetric morphogen expression in regenerating male sail feathers and used transcriptome and H3K27ac ChIP-seq to uncover male and female signaling pathways and regulatory elements. Comparative epigenomic profiling reveals enriched estrogen receptor binding motifs in females. Hormone profiling shows seasonal variation, with a marked rise in female estrogen levels preceding the mating season. These results imply Mandarin duck sail feathers integrate local morphogenetic programs, epigenetic regulation, and systemic hormonal cues to orchestrate sexually dimorphic and seasonally dynamic feather morphogenesis. This work establishes a framework for further mechanistic study of the interplay between regeneration, regional identity, and hormonal plasticity in a vertebrate integumentary system.

**Supplementary Information:**

The online version contains supplementary material available at 10.1038/s41598-025-20446-3.

## Introduction

The integument serves as a dynamic interface between organisms and their environment, requiring continuous adaptability for survival and reproduction. In amniotes, integumentary appendages, like hairs, feathers, and scales, arise from a shared pool of stem cells^1^, enabling cyclic renewal and continual morphogenesis in response to extrinsic environmental changes such as seasonal variations^2^. This adaptability is achieved through three key principles: periodic patterning for appendage generation, stem cell-based follicle renewal, and spatiotemporal specificity in appendage phenotypes^3–6^. Research on mammals, including humans, demonstrate that hair follicles can exhibit distinct cycling behaviors and characteristics that vary by region and age, influenced by hormonal and seasonal cues^6,7^ underscoring the integument’s utility as a model for studying post-embryonic developmental plasticity linked to stem cell regulation.

Birds exhibit remarkable diversity in feather phenotypes, making them ideal for investigating the regulation of these changes. Their feather follicles regenerate cyclically, yielding varied forms, colors, and rigidities that differ by body regions (natal, juvenile, adult). The primary developmental transition occurs post-hatching, replacing radially symmetric natal down with region-specific juvenile feathers after the first molt^8^. A series of secondary transitions provide further variations using specific regional factors and regulatory mechanisms, including morphogen gradients^4,9^, epigenetic switches^10^, and hormonal influences^11^, that orchestrate these transformations.

The Mandarin duck (*Aix galericulata*) exemplifies spectacular plumage changes, with downy and then juvenile feathers showing similarities between sexes, followed by seasonal pronounced sexual dimorphism at maturity^12^. Males develop extravagant feather morphologies and coloration, particularly during breeding season, while females retain juvenile characteristics. This is particularly notable for sail feathers, located at secondary remex 12 (the 12th secondary flight feather) on each wing^12^. This sexual dimorphism in feather morphology presents an excellent opportunity to investigate the genetic and hormonal drivers of cyclical, sex-specific feather development.

In this study, the Mandarin duck sail feather is proposed as a model for understanding lifelong developmental modulation. Here we explore the roles of morphogen expression as well as transcriptomic and epigenetic changes underlying these seasonal region-specific sexual dimorphisms by comparing male and female feathers located at remex11 (flight feathers) and remex 12 (sail feathers). We also explore the expression of genes in male Mandarin ducks that we previously showed are important in the formation of asymmetric feathering in chickens^4^. Indeed, the research reveals seasonal morphological changes in sail feathers and identifies asymmetric morphogen expression patterns in regenerating male feathers. Through RNA-seq and H3K27ac ChIP-seq analyses, sex-specific regulatory programs linked to WNT, retinoic acid and Sonic hedgehog (SHH) signaling pathways were uncovered in males, alongside female-specific enhancer elements associated with estrogen receptor motifs in females. Additionally, circulating sex hormone levels were monitored over 42 months, highlighting seasonal estrogen peaks before feather molts. This comprehensive framework illustrates how diverse feather forms arise from a shared stem cell population, influenced by both localized and systemic regulatory factors. Although further investigation is needed to establish causal relationships, this study establishes the Mandarin duck as a suitable model for examining lifelong integumentary plasticity.

## Results

### Multi-layered environmental regulations contribute to the extraordinary morphogenesis of asymmetric forms and colors in male Mandarin Duck sail feathers

**Fig. 1:**
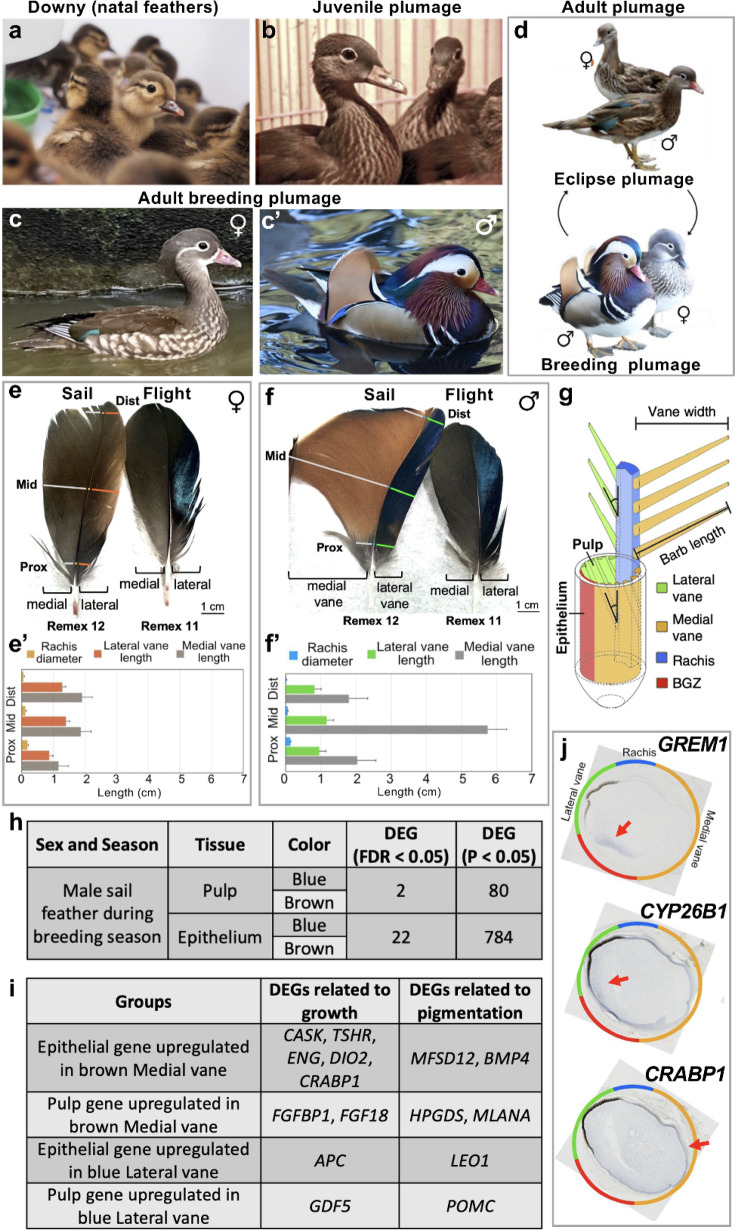
The extraordinary feather cycling of Mandarin ducks and differentially expressed genes in male Mandarin duck sail feathers. (**a-c**,** c’**) The plumage of duckling (**a**), juvenile (**b**) and sexually dimorphic adult males (**c**) and females (**c’**). (**d**) The molting cycle of eclipse plumage (top) and breeding (bottom) plumage. Seasonal photoperiod variation leads to feather molting and subsequent replacement. In adult males, this allows for colorful feathers in winter and spring followed by muted colors in summer and fall. (**e**,** f**) Images showing the adult male and female sail feather (the 12th secondary flight feather, remex 12) and the neighboring secondary flight feather (remex 11) during breeding season. The width of proximal (Prox), middle (Mid) and distal (Distal) asymmetric lateral blue and medial brown vanes were measured. (**e’**,** f’**) The average lateral blue and medial brown vane lengths and rachis thickness were calculated with standard deviations for females (*n* = 6) in (**e’**) and males (*n* = 12) in (**f’**). (**g**) Schematic drawing of feather follicle^4^. BGZ (barb generative zone). (**h**) DEGs between the male sail feather medial brown and lateral blue vanes were found in the epithelium and pulp. (**i**) DEGs known to regulate feather asymmetry or pigmentation. (**j**) In situ hybridization performed only on asymmetric male sail feathers during breeding season showing the distributions of *GREM1*, *CYP26B1* and *CRABP1*. Locations of the rachis (blue), lateral blue vane (green), medial brown vane (yellow) and barb generative zone (red) are indicated. Arrows point to positive signals.

Mandarin duck (*A. galericulata*) downy, juvenile, and adult plumage development was observed across different life stages (Fig. 1a-d, c’). Initially, young males and females exhibit similar downy feathers (Fig. 1a). During the primary transition to juvenile feathers, regional characteristics develop, although these remain largely comparable between sexes (Fig. 1b). Then secondary transitions in adult male plumage produce annual cycles of eclipse and breeding phases (Fig. 1d), where distinct sexual dimorphisms emerge (Fig. 1c, c’). Adult breeding season male and female sail (remex 12) and adjacent flight (remex 11) feathers are shown (Fig. 1e, f). Male sail feathers emerging on each side of the wing midline at remex 12 (sail feather) are notably enlarged and asymmetrical in size, shape, and color compared to remex 11 (secondary flight feather; Fig. S1). Male sail feathers play an important role in attracting a female mate ^[12]^ (Fig. 1f). Female feathers in equivalent positions maintain their smaller, symmetrical feathers which retain cryptic patterns (Fig. 1e). To characterize the asymmetric sail feather form, we compared rachis diameter and barb length of female (Fig. 1e’; *n*=6) and male feathers (Fig. 1f’; *n*=12) along their lateral blue and medial brown branches (Fig. 1e, f). The rachis was thickest in the proximal region (males: 0.14 +/- 0.03 cm, females: 0.18 +/- 0.04 cm in diameter, 1.29 times higher in females) and tapered distally (males: 0.02 +/- 0.02 cm, females: 0.05 +/- 0.02 cm, 2.5 times higher in females). Lateral blue vane branches showed slight variations in length from proximal (males: 0.96 +/- 0.1 cm, females: 0.87 +/- 0.11 cm, 1.1 times higher in males) to middle (males: 1.17 +/- 0.10 cm, females: 1.40 +/- 0.12 cm, 1.25 times higher in females) to distal (males: 0.82 +/- 0.20 cm, females: 1.28 +/- 0.07, 1.56 times higher in females) regions. In contrast, medial brown vane branch length varied widely between males and females from the proximal (males: 1.80 +/- 0.40 cm, females: 1.16 +/- 0.15, 1.55 times higher in males) to middle (males: 5.75 +/- 0.50 cm, females: 1.87 +/- 0.20 cm, 3.075 times higher in males) to distal (males: 2.04 +/- 0.50 cm, females: 1.91 +/- 0.33, 1.07 times higher in males) regions (Fig. 1e, f’). Multi-layered environmental regulations contribute to sexually dimorphic fan-shaped morphology.

### Asymmetric morphogen expression in Mandarin Duck sail feathers

To explore the molecular mechanisms for asymmetry in breeding season male sail and secondary flight feathers, we conducted a transcriptome analysis on dissected medial brown and lateral blue vane epithelium and dermis using two replicate biological samples for each of the 4 types of tissue (Fig. 1f, g). This analysis revealed 80 (Table S2) differentially expressed genes (DEGs) in the dermal pulp and 784 DEGs (Table S1) in the epithelium (*p* < 0.05) (Fig. 1h). The epithelium showed a log fold change (logFC) ranging from 7.68 for the medial brown vane to -6.96 for the lateral blue vane (Table S1), with gene ontology terms indicating involvement of aldosterone signaling, protein ubiquitination, and RhoA signaling, unfolded protein response, and Ataxia-Telangiectasia and Mantle Cell Lymphoma signaling. In the pulp, the logFC varied from 3.81 to -7.43 (Table S2). The top 5 gene ontology terms were tight junction signaling, agranulocyte adhesion and diapedesis, clathrin-mediated endocytosis signaling, osteoarthritis pathway, and actin cytoskeleton signaling.

In the medial brown and lateral blue epithelium and mesenchymal pulp, genes controlling growth may lead to feather asymmetries (Fig. 1i). Elevated genes in the medial brown epithelium (Table S1) include *CASK* (Calcium/calmodulin-dependent serine protein kinase; LogFC = 5.68), *TSHR* (thyroid-stimulating hormone–receptor; logFC = 5.11), *ENG* (Endoglin; logFC = 4.25), *DIO2* (Iodothyronine Deiodinase 2; logFC = 3.81), and *CRABP1* (Cellular retinoic acid binding protein 1; logFC = 2.53) (Table S1). In mice, nuclear CASK regulates keratinocyte proliferation in basal epithelial cells and hair follicles^13^. The binding of Thyroid stimulating hormone to TSHR and ENG induces cultured human skin keratinocytes and fibroblasts to proliferate^14,15^. Genes elevated in lateral blue vane epithelium include *APC* (Adenomatous polyposis coli; logFC = -2.12), which is essential for hair development^16^ and may inhibit β-catenin-mediated cell proliferation, potentially leading to shorter barb lengths. Within the medial brown pulp (Table S2), *FGFBP1* (Fibroblast growth factor binding protein 1, logFC = 3.32) and *FGF18* (Fibroblast growth factor 18; logFC = 2.71), which are associated with keratinocyte proliferation in mouse and human skin, are expressed. FGF18 suppresses the transition of murine hairs from telogen to anagen^17–20^. Down-regulation of FGF18 with siRNA promotes hair growth^21^. FGFBP1 controls FGF activity by carrying FGFs to their appropriate receptors^22^. In feathers, FGFs promote feather production while configuring the proximal-distal feather axis^23,24^. The lateral blue vane pulp expresses *GDF5* (Growth differentiation factor 5; logFC = -6.42). GDF5 promotes cultured mesenchymal stem cells to differentiate into extracellular matrix-forming fibroblasts^25^.

 Next, we evaluated genes underlying differences in medial-lateral vane pigmentation (Fig. 1i). In the medial brown vane, epithelial genes (Table S1) include *MFSD12* (Major facilitator superfamily domain containing 12; logFC = 5.01) and *BMP4* (Bone morphogenetic protein 4; logFC = 2.36). BMP4 via interactions with Agouti and MFSD12 regulate pheomelanin synthesis^26,27^ to produce brown pigments. The lateral blue vane epithelium expresses *LEO1* (Leopard; log FC = -2.19) encoding Connexin 41.8 which promotes xanthophore – melanophore interactions^28^ that regulate zebrafish pigment stripe patterning^29^. In the medial brown vane dermal pulp, genes affecting pigmentation (Table S2) include *HPGDS* (Hematopoietic Prostaglandin D Synthase; logFC = 3.59) and *MLANA* (*MART-1*, melanoma-associated antigen recognized by T‐cell 1; logFC = 2.73). HPGDS potentially influences pigmentation^30^ and MLANA serves as a marker for early-stage melanogenesis^31^. The lateral blue vane’s dermal expression of *POMC* (Pro-opiomelanocortin; logFC = -5.50) encoding an α‐melanocyte‐stimulating hormone (α‐MSH) precursor, impacts eumelanin and pheomelanin formation through MC1R (Melanocortin 1 receptor)^32^. POMC and MC1R have also been reported in chicken feathers^33^. This molecular framework elucidates the molecules behind the observed color differences in feathers.

We previously examined the spatial expression profile of several previously identified genes contributing to chicken asymmetric flight feather formation^4^. Feathers initially grow as cylinders and open at the site of the barb generative zone (BGZ). For symmetric feathers, the barb generative zone is opposite to the rachis and both barbs are of equal length. For asymmetric feathers, the barb generative zone is off center, so one branch is long while the other is short (Fig. 1g). Here, we examine their expression in breeding season male Mandarin duck sail feathers using in situ hybridization to localize factors involved in asymmetry. These genes include *GREM1* (Gremlin 1), *CYP26B1* and *CRABP1* (Fig. 1j). Although *GREM1* and *CYP26B1* were not detected as DEGs in our transcriptome data (Tables S1 and S2), we suspect this is because the libraries used for RNA-seq were from whole developing feather and the relatively small area occupied by these genes was insufficient to be recognized. *GREM1*, a BGZ marker, is expressed toward the lower feather region, opposite to the rachis (arrow in Fig. 1j, top panel). *CYP26B1*, encoding a retinoic acid-degrading enzyme, is expressed lateral to *GREM1* (arrow in Fig. 1j, middle panel). *CRABP1* translocates retinoic acid to the nucleus and is upregulated in the medial brown vane epithelium (Fig. 1j, bottom panel). The combinatory effect of CRABP1 and CYP26B1 was postulated to create a retinoic acid gradient that facilitates the lateral expansion of GREM1 expression, which promotes the extreme medial brown vane asymmetric phenotype^4^.

### Differences of epigenetic profiles between male and female sail feathers analyzed by H3K27ac ChIP-seq

**Fig. 2:**
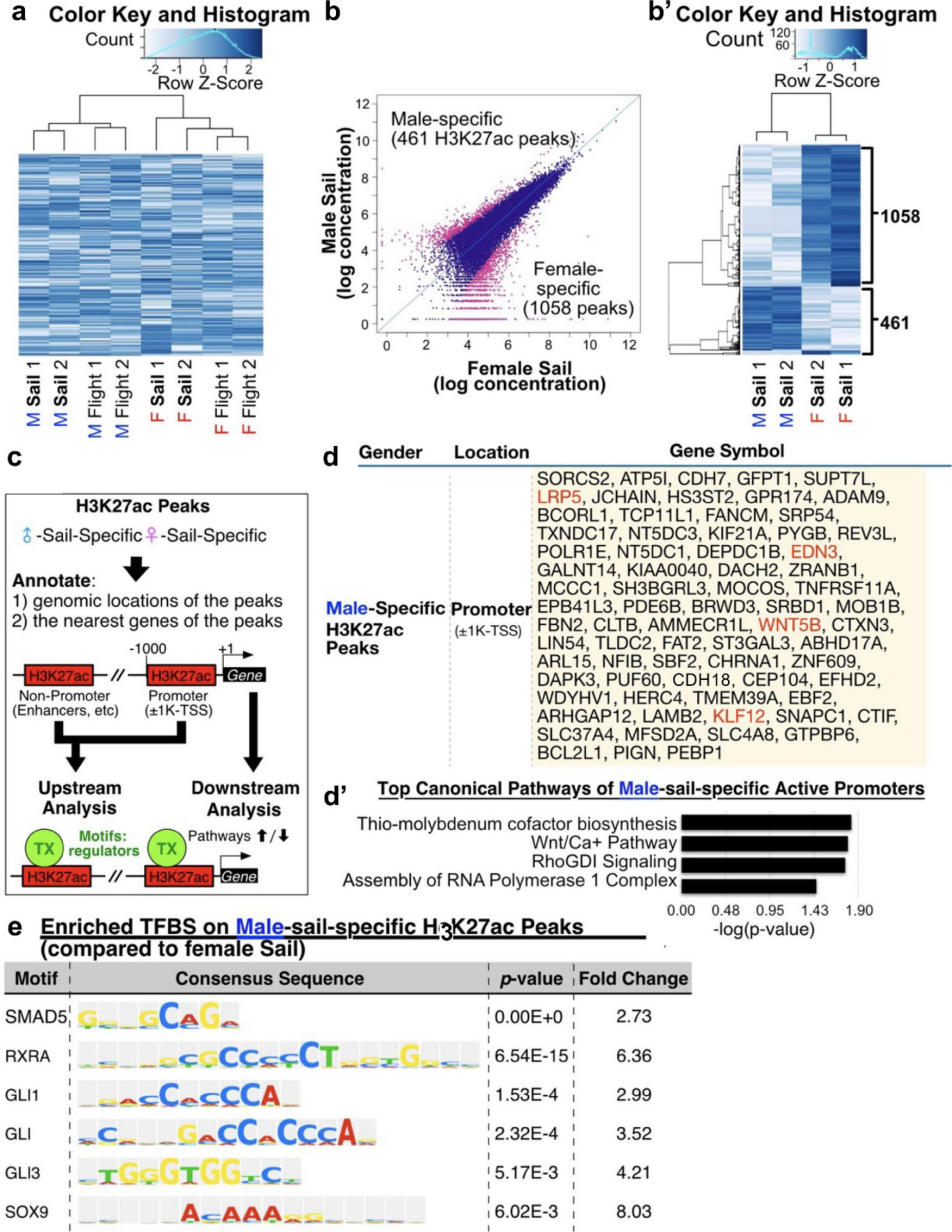
Analyzing sexually dimorphic sail feathers by comparing H3K27ac ChIP-seq data in male and female sail feathers. (**a**) Differentially enriched H3K27ac peaks (FDR < 0.05) between duplicate samples of male and female sail feathers (remex 12) and flight feathers (remex 11). (**b**,** b’**) Focusing exclusively on male and female sail feathers (remex 12), 1,058 peaks are present in female and 461 peaks are present in male sail feathers. (**c**) Diagram showing H3K27ac peaks indicate enhancers or promoters that drive expression of male-specific and female-specific peaks. From this data we can examine downstream gene expression by focusing on genes nearest to the peaks and also on upstream regulators by examining enriched transcription binding site motifs. (**d**) List of male-specific downstream genes expressed in our dataset that are closest to the H3K27ac peaks. Genes discussed are highlighted in red. (**d’**) Top canonical pathways identified in the analysis of male sail feather-specific peaks. (**e**) Enriched transcription factor binding sites found in the male sail feather-specific H3K27ac peaks.

To identify the upstream regulation of male and female sail feather (remex 12) and secondary flight (remex 11) development, chromatin immunoprecipitation sequencing (ChIP-seq) against Histone H3 lysine 27 acetylation (H3K27ac), an active chromatin signature^34^, was performed on epithelial and mesenchymal (pulp) feather follicle components (Fig. 2a). Principal component analysis confirmed the consistency among the eight samples consisting of two biological replicate samples for male and female sail (remex12) and flight (remex11) feathers (Fig. S2). The ChIP-seq result identified sites of active promoters or enhancers on male or female sail (remex 12) feathers that might be involved in sexually dimorphic asymmetric feather shape and color. Differential peak analysis found 461 male-sail-specific H3K27ac peaks and 1,058 female-sail-specific H3K27ac peaks (Fig. 2b, b’). Genes whose transcription start site (TSS) fall within 1 kb of these peaks are likely downstream targets regulated by these promoter regions (Fig. 2c). These data show that in males, WNT/Calcium signaling (Fig. 2d, genes highlighted in red) and other pathways are up-regulated. Ingenuity Pathway Analysis (IPA) suggests that pathways enriched in males include Thio-Molybdenum Cofactor Biosynthesis (increases translation accuracy), WNT/Ca^2+^ pathway (organismal growth and development), RhoGDI signaling (activating guanine exchange factors affecting multiple aspects of cell behavior), and Assembly of RNA Polymerase I Complex (transcription regulation) (Fig. 2d’). For females, genes near promoters include Melatonin Degradation (seasonal rhythms), Caveolar-mediated Endocytosis Signaling (membrane trafficking), Assembly of RNA Polymerase III Complex (transcription regulation), and Glycogen degradation II and III (endocrine system development and function). Enriched transcription factor binding motifs lying upstream of H3K27ac peaks in male-specific sail feathers suggest β-catenin/WNT signaling (growth control), TGFβ, retinoic acid (control growth and differentiation), SHH (control growth, differentiation and patterning) and *SOX9* (control growth and homeostasis) might play a role in modulating downstream gene expression (Fig. 2e) and have profound effects on the growth, differentiation, and patterning of feather morphogenesis leading to seasonal dimorphisms^35–38^.

### Differences of epigenetic profiles between sail and adjacent flight feathers analyzed by H3K27ac ChIP-seq

**Fig. 3:**
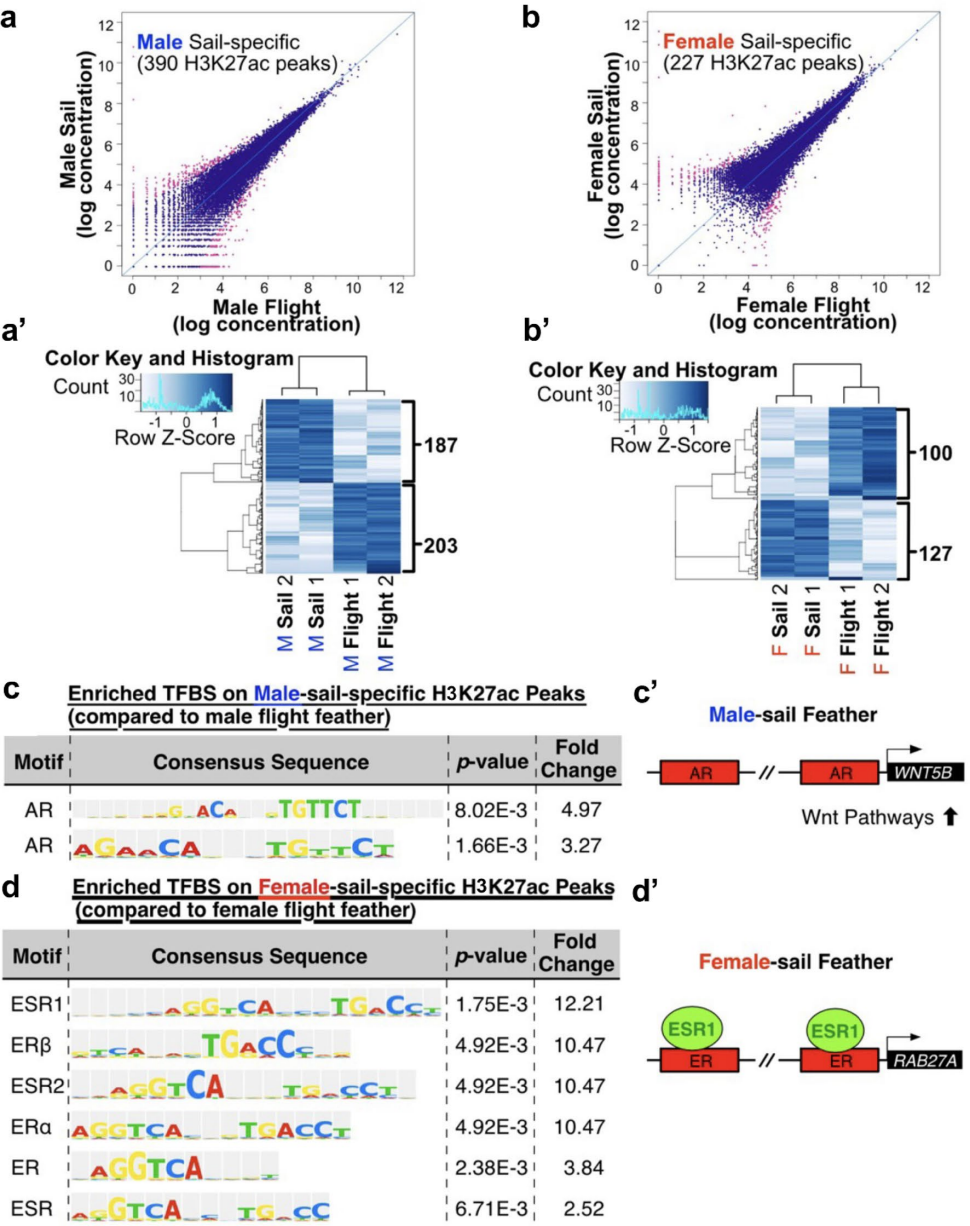
Comparison of H3K27ac ChIP-seq data, an active chromatin signature, between sail feathers (remex 12) and adjacent flight feathers (remex 11) in male and female Mandarin ducks. (**a)** Male sail feathers (remex12) compared to adjacent flight feathers (remex 11) show 390 H3K27ac peaks (FDR < 0.05). **(a’**) Among these 390 H3K27ac peaks, 187 are elevated in sail feathers (remex 12) and 203 are elevated in adjacent flight feathers (remex 11). (**b)** Female feathers (remex 12) compared to adjacent flight feathers (remex 11) have 227 H3K27ac peaks (FDR < 0.05). **(b’**) Among these 227 peaks, 127 are elevated in remex 12 and 100 are elevated in adjacent flight feathers (remex 11). (**c**) Enriched transcription factor binding sites (TFBS) in male sail feathers (remex 12) versus adjacent flight feathers (remex 11) show elevated binding to the Androgen Receptor (AR) in sail feathers. (**c’**) In male sail feathers, the AR binds close to the gene encoding Wnt5B which is involved in growth control. Schematic diagram of our data suggesting that binding to the AR leads to increased Wnt pathway activation. (**d**) Transcription factor binding site peaks elevated in female remex 12 versus remex 11 feathers show an enrichment for the Estrogen Receptor (ER) in remex 12. The ER binds close to the gene encoding RAB27A which leads to melanocyte transport into keratinocytes. (**d’**) Schematic diagram of our data suggesting that in females binding to the Estrogen receptor leads to increased RAB27A activation.

We further focus on body region gene expression differences by comparing sail feathers (remex 12) with neighboring secondary flight feathers (remex 11) in both males and females. In males, 390 peaks were identified. Hierarchical clustering showed 187 genes were enriched in sail feathers and 203 in male secondary flight feathers (FDR < 0.05) (Fig. 3a, a’). In females, a total of 227 peaks were identified with 100 peaks enriched in female sail feathers and 127 enriched in secondary flight feathers (Fig. 3b, b’).

We then explored the enriched gene binding motifs in promoter regions of sail and secondary flight feathers, revealing a significant enrichment of androgen receptor (AR) in male sail feathers compared to flight feathers (p < 0.01, motif occurrence fold change > 2) (Fig. 3c and Table S3). Previous research indicates that activation of androgen pathways can induce hair growth in sexually mature adults^11^. The localization of AR binding motifs near the *WNT5B* gene (Fig. 3c’) correlates with a potential role for WNT signaling, which has been implicated in growth control^8^, to play a role in the development of male sail feather morphogenesis.

In female sail versus flight feathers, there is an enrichment of Estrogen receptors: (ESR1 (Estrogen receptor 1), ESRβ (Estrogen receptor β, ESR2 (Estrogen receptor 2), ERα (Estrogen receptor α), ER (Estrogen receptor) and ESR (Estrogen receptor)) (p < 0.01, motif occurrence fold change > 2) (Fig. 3d and Table S4). The binding motifs for ESRα and ESR1 are nearly identical as are the binding motifs for ESRβ and ESR2. The binding motifs for ER and ESR represent the common motifs for each of the estrogen receptors. Unlike androgens, estrogens suppress hair growth^39^. The estrogen receptor binding motif is located near the *RAB27A* gene, which is involved in melanosome binding to melanocytes for subsequent transfer to keratinocytes (Fig. 3d’)^40^. *RAB27A* expression correlates with a potential role in forming the lateral blue pigmentation observed in female remex 12 feathers.

### Seasonal variations in Circulating hormone during the breeding season in Mandarin ducks

**Fig. 4:**
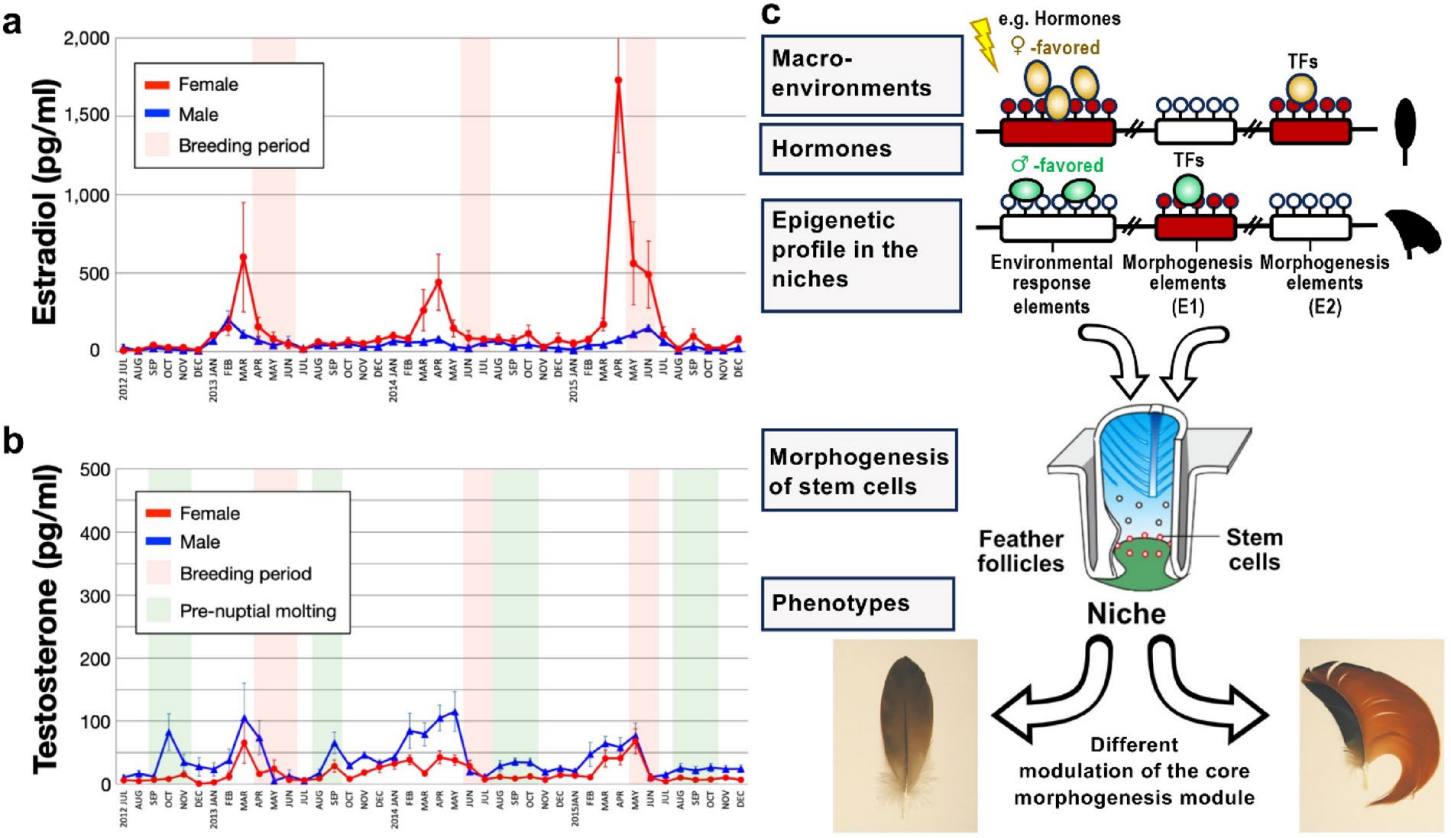
The development of sexually dimorphic feathers under the circulating hormone of Mandarin ducks. (**a**,** b**) Monthly measurements of circulating (**a**) estradiol and (**b**) testosterone in males (blue) and females (red) over a 42-month period. Breeding and egg-laying seasons are indicated (pink bands). Times of pre-nuptial molts (the left green band) and breeding periods (green) also are indicated. (**c**) Conceptual diagram showing how we perceive the multi-scale regulation of asymmetric sail feather morphogenesis during breeding season. Macro-environmental input may be interpreted as hormone levels that act on feather follicles. For instance, yellow ovals represent hormones from females while green ovals represent hormones from males. Epigenetic profiles of morphogens in follicles are different in male and female mandarin ducks (red versus white rectangle regions). Sex-favored transcription factors (TFs) (yellow versus green circles) bind to different morphogenesis elements (E2 versus E1). Therefore, morphogen expressions are different in the micro-environmental niches, guiding stem cells to form sexually dimorphic sail feather phenotypes (symmetric female sail feather versus asymmetric male sail feather). For the hormones and epigenetic profiles, we have identified candidates that are associated with the phenotypes in this study, paving the way for future causal experiments.

To investigate the role of sex hormones in sex-dimorphic plumage, blood samples were acquired monthly from hens and drakes. Serum estradiol (Fig. 4a) and testosterone (Fig. 4b) levels were charted over 42-month period using the enzyme-linked immunosorbent assay (ELISA). Female estradiol levels peaked in March-April and remained elevated through June (Fig. 4a, red line), with distinct breeding periods identified following these peaks (Fig. 4a, pink bars). In contrast, male estradiol levels remained low with minor increases (Fig. 4a, blue line).

Testosterone levels exhibit seasonal variations, with female peaks occurring in March-May in different years (Fig. 4b, red line). While males also peak in these months, they have minor peaks in the fall that lead to breeding plumage development (Fig. 4b, blue line). Male drakes experience a testosterone peak prior to a fall molt, leading to breeding plumage development (Fig. 4b, green bars). Post-breeding, both sexes see a testosterone rise in late winter to spring, followed by a molt that results in eclipse plumage. These hormone level trends underscore the role of hormones in molting and the development of distinct male and female sail feathers. However, potential influencing environmental conditions (photoperiod, temperature, diet) that may influence hormone levels and molt timing have not yet been explored.

## Discussion

In this study, we develop a new animal model, the Mandarin duck, to investigate the regulatory mechanisms controlling sexual, seasonal and regional skin appendage morphology and pigmentation patterning. Remex 12, a secondary flight feather on each side of male Mandarin ducks, becomes an enlarged, asymmetric and brightly colored sail feather during mating season. It returns to a more symmetric feather with a muted pigmentation pattern outside of the mating season. Female feathers at remex 12 and male feathers at remex 11 maintain a small size with muted pigmentation throughout the year. Hence, feather follicle stem cells can form different feather phenotypes (form and color) that are modulated by sexual, seasonal and regional cues.

Our findings suggest that seasonal sexual dimorphism in sail feathers among males and females is influenced by hormone – receptor driven transcription factor activity (Fig. 4c). Systemic levels of androgens and estrogens peak before the breeding season, potentially influencing morphological and pigmentation features in specific regions of male and female birds. Hormone receptors are known to exhibit sex-dependent expression patterns and vary in their expression by region and timing. Notably, while progesterone and estrogen receptors are present during feather and skin growth, progesterone receptor levels decrease during barbule differentiation, whereas estrogen receptors persist^41^. Previous research indicates that estrogens enhance female feathering, whereas androgens do not promote male feathering^11,42–46^. Male ducks exhibit enlarged and asymmetric sail feathers due to androgen receptor activation of WNT, retinoic acid, and sonic hedgehog pathways, while female ducks produce eumelanin under estrogen receptor influence.

The cyclic regeneration of feathers offers insights into how epigenetic mechanisms can facilitate morphogenetic changes through the influence of sex hormones and seasonal variations^3,47,48^. Our results suggest that remex 12 feathers exhibit enriched sex hormone transcription binding sites in comparison to adjacent flight feathers, contributing to the observed seasonal variations of sexually dimorphic characteristics in sail feathers. In males, the medial brown branches express higher levels of genes involving epithelial growth (CASK, TSHR and ENG) that promote longer feather branches than the lateral blue side, while CRABP1 helps to establish the retinoic acid gradient guiding feather asymmetry. In contrast, the lateral blue branches express *APC* and *FGF18*, which may suppress epithelial proliferation. Differences in medial brown and lateral blue pigmentation are controlled by pheomelanin inducing genes (*MFSD12* and *BMP4*) on the medial side and eumelanin producing genes (*HPGDS*,* MLANA*, and *POMC*) on the lateral side.

Furthermore, H3K27ac ChIP-seq shows male sail feathers are significantly influenced by androgen receptors. Androgen receptor binding sites are located near WNT5B, suggesting WNT signaling is up-regulated in male sail feathers. The androgen signaling in barn owls promotes pheomelanin expression and suppresses eumelanin^49^, aligning with the distinctive coloration and size of sail feathers. In contrast, female sail feathers show significant involvement of estrogens. Estrogen receptor binding motifs are located near *RAB27A*, which might promote the darker pigmentation in female remex 12 feathers. While Tissue-specific DNA methylation may epigenetically regulate region-specific ESR1 expression in humans^50^.

Our findings indicate that androgen and estrogen receptors play crucial roles in feather morphogenesis and pigmentation. These findings lay the groundwork for further functional investigations into the regulatory frameworks underlying sexually dimorphic-, season- and region-specific feather morphogenesis. Differential expression of transcription co-factors may further influence regional cell sensitivity to hormonal signals.

### Limitations of the study and future works

Due to the limited number of sail feathers per Mandarin duck, we could not collect sufficient sample numbers to comprehensively assess the gene distribution identified in our RNA-seq and ChIP-seq studies across various developmental stages. While we aimed to analyze and compare the morphology of male and female sail (remex 12) and adjacent flight feathers (remex 11) during both breeding and non-breeding seasons, logistical challenges, including the availability of specimens and weather fluctuations, hindered our efforts. This also precluded our ability to perform functional tests on our genes of interest. We plan to continue sample collection and development of this research in future studies.

## Materials and methods

### Ethics statement and animals

All the Mandarin ducks (*A. galericultata*) used in this study were from the National Chung Hsing University (NCHU; Taichung, Taiwan) poultry farm. All experimental protocols were approved by the NCHU Institutional Animal Care and Use Committee (IACUC No. 103-43^R^). All methods were carried out in accordance with relevant guidelines and regulations. All methods were reported in accordance with ARRIVE guidelines.

### Measurements of rachis diameter and Vane widths

Male and female remex 12 (sail feathers) and remex 11 (flight feathers) were collected during breeding season. The rachis diameter and vane widths were measured in the regions indicated by the green and white lines. These represent the proximal, distal and middle point (the site of maximal width). Rachis diameter was measured with a micrometer at 20x magnification. Vane widths were measured without microscopic enlargement. All measurements for sail (remex 12) and flight (remex 11) feathers were collected from the same bird. We collected samples from 12 male and 6 female birds. Lateral vanes are the outer vanes found at the leading edge of an asymmetric sail or flight feather. Medial feather vanes are the inner vanes found at the trailing edge of a sail or flight feather.

### Paraffin section and in situ hybridization

Regenerating male sail feathers were plucked and fixed in 4% paraformaldehyde at 4 °C overnight, then serially dehydrated in alcohol, cleared in xylene, and embedded in paraffin. Cross sections were cut at 8 μm. Section in situ hybridization was performed as described^51^. Sections were rehydrated, acetylated and incubated with digoxigenin labelled probes overnight at 65 °C. Primers used to make the probes: d-GREM1-S: GCTATCCCTCCTCCTGACAAG, d-GREM1-AS: CTTCCTAGGGGGCTGAAGCTC; d-CRABP1-S: GTCAACGCCATGCTCAGGAA, d-CRABP1-AS: CGGTCACATACAACACCGCA; d- CYP26B1-S: TGTTGTGGGGCTAAGCAGAC, d-CYP26B1-AS: GCATAGTCCTTGCCCTGTGT. After washing, samples were incubated with anti-digoxigenin-AP secondary antibodies at 4 °C overnight. Color was detected using the Promega NBT/BCIP substrate.

### RNA sequencing and data analysis

To gather transcriptome profiles from male sail and flight feathers, we plucked feathers in two regions (remex 11 and 12 of both wings) from two male individuals during breeding season. Then, these four regenerated feathers were collected around four weeks and dissected to separate epithelium and pulp before RNA extraction.

In the eight samples, each group had two biological replicates. Total RNAs were extracted using the standard Trizol extraction protocol (Cat #15596026, Invitrogen). 1 µg of total RNA from each sample was used to construct an RNA-seq library using the TruSeq RNA Prep Kit v2 (Cat #RS-122–2001/-2002, Illumina). Libraries were prepared from epithelium and mesenchyme using the standard protocol^52^. Sequencing (75 nucleotide single read) was performed using Hi-seq 2000 or NextSeq 500 platform at the USC Molecular Genomic Core (MGC). The alignment, quantification, normalization, and differential expression analysis were performed by STAR 2.4.1d^53^, HTSeq-count 0.6.0^54^, TMM^55^, and edgeR^56^, respectively.

### Gene ontology analysis

The pathway enrichment analysis was generated through the use of Ingenuity Pathway Analysis (IPA, QIAGEN Inc.) and Partek Genomics Suite software, version 6.6, build 6.14.0514 (Partek Inc.).

### Chromatin Immunoprecipitation

To collect ChIP-seq samples from sail and flight feathers, we plucked feathers from two regions (remex 11 and 12 of both wings) of four individuals (two males and two females) during breeding season. Then, these eight regenerated feathers (approximately 4–5 cm in length) were collected four weeks after plucking.

We employed ChIP-seq to map whole-genome chromatin signatures with antibody to histone acetylation: H3K27ac (ab4729, Abcam). We collected samples from the epithelium and mesenchyme at 4 weeks after plucking. Input DNA was used as background controls. For ChIP-Seq analysis, we used bowtie2^57^, macs2^58^, bedtools, and deepTools^53^ for alignment, peak calling, manipulation, and visualization, respectively. Motif Prediction was determined using the HOMER findMotifsGenome command^59^. Identification of differential ChIP-seq peaks was determined using DiffBind (differential binding analysis) using Score Column = 5; FDR Threshold = 0.05. Peaks were mapped to the Mandarin duck genome. In total, eight samples were sequenced and analyzed for H3K27ac ChIP-seq.

### ELISA

Blood testosterone and estradiol levels were detected by ELISA assays following the manufacturer’s instructions (Item NO.582701 for T and Item NO.582251 for E2, Cayam, MI, USA). Blood was taken over the course of 42 months from 5 to 14 male and 5–10 female Mandarin ducks as birds were lost and replaced. Briefly, serum was obtained by centrifuging the collected blood at 3,000 rpm for 15 min at 4 °C. 50 µl of sample or standard serum in duplicate were analyzed. Once the tracer and antiserum were mixed, the ELISA plates were rotated at room temperature for 1 h (E2) or 2 h (T). The plates then were washed and placed in a dark, shaking incubator with Ellman’s reagent at room temperature for 60–90 min. Data was acquired with an ELISA plate reader at wavelengths between 405 and 420 nm. Hormone levels were calculated in relation to a standard concentration curve.

## Supplementary Information

Below is the link to the electronic supplementary material.

Supplementary Material 1

Supplementary Material 2

Supplementary Material 3

Supplementary Material 4

Supplementary Material 5

## Data Availability

The Mandarin duck reference genome ^60^ was deposited in NCBI under BioProject accession PRJNA787744. The next-generation sequencingdatasets generated in this study have been deposited in the NCBI Gene Expression Omnibus (GEO). Bulk RNA-seq data are available underaccession GSE308466, and H3K27ac ChIP-seq data are available under accession GSE308467. All datasets will be publicly accessible uponpublication.
