# Supplementary material for: Sexually dimorphic sail feathers in the Mandarin duck as a model for lifelong developmental modulation
Source: Sci Rep. 2025 Oct 21;15:36593. doi: 10.1038/s41598-025-20446-3 (PMC12540880; doi:10.1038/s41598-025-20446-3)
Supplement: Supplementary file 5 — Supplementary Material 5 [file 41598_2025_20446_MOESM5_ESM.pdf]

Fig. S1

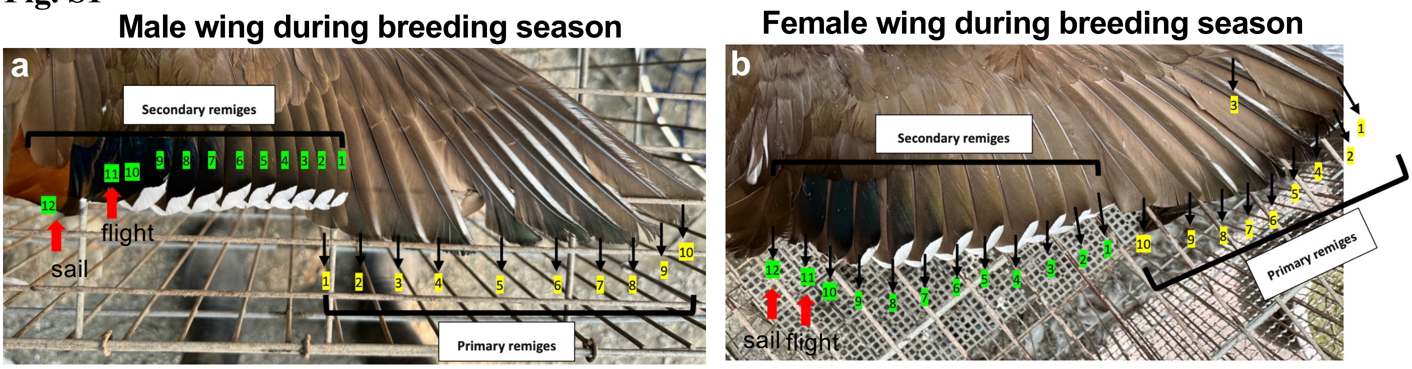

Fig. S1. The positions and morphology of secondary flight feathers in the Mandarin ducks used in this study. Ten primary flight feathers (#1-10, yellow) and twelve secondary flight feathers (#1-12, green) on one side of each wing were numbered. Male (a) and female (b) wing plumage during breeding season. The samples collected in this study (indicated by arrows) include the "sail feather" from the 12th secondary remex (remex 12) and "flight feather" from the 11th secondary remex (remex 11).

Fig. S2

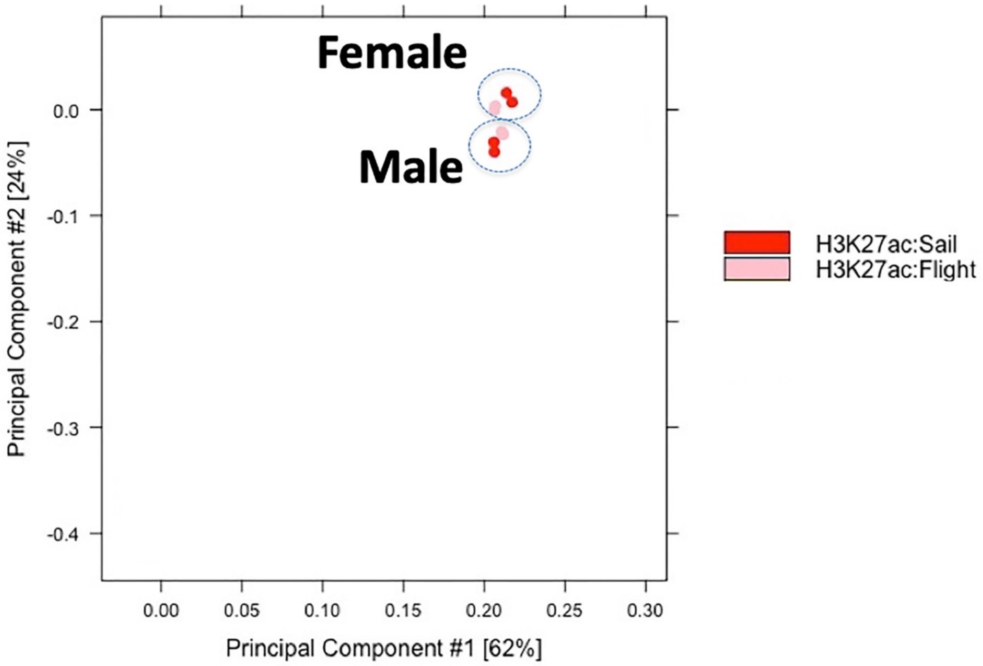

Fig. S2. Principle component analysis (PCA) of H3K27ac ChIP-seq data for male vs. female sail and flight feathers. Four groups of samples (female sail, female flight, male sail, and male flight feathers) were analyzed, with two biological replicates for each group. Sail of flight feathers were not clustered together; instead, feather tissue from females and males are separated by dashed lines.
